# Comparative Access to and Use of Digital Breast Tomosynthesis Screening by Women’s Race/Ethnicity and Socioeconomic Status

**DOI:** 10.1001/jamanetworkopen.2020.37546

**Published:** 2021-02-19

**Authors:** Christoph I. Lee, Weiwei Zhu, Tracy Onega, Louise M. Henderson, Karla Kerlikowske, Brian L. Sprague, Garth H. Rauscher, Ellen S. O’Meara, Anna N. A. Tosteson, Jennifer S. Haas, Roberta diFlorio-Alexander, Celia Kaplan, Diana L. Miglioretti

**Affiliations:** 1 Department of Radiology, University of Washington School of Medicine, Seattle; 2 Department of Health Services, University of Washington School of Public Health, Seattle; 3 Kaiser Permanente Washington Health Research Institute, Seattle, Washington; 4 Huntsman Cancer Institute, Department of Population Health Sciences, University of Utah, Salt Lake City; 5 Department of Radiology, University of North Carolina at Chapel Hill; 6 Department of Epidemiology, University of North Carolina at Chapel Hill; 7 Department of Medicine, University of California, San Francisco; 8 Department of Epidemiology and Biostatistics, University of California, San Francisco; 9 General Internal Medicine Section, Department of Veterans Affairs, University of California, San Francisco; 10 Department of Surgery, University of Vermont Cancer Center, Burlington; 11 Department of Radiology, University of Vermont Cancer Center, Burlington; 12 Division of Epidemiology and Biostatistics, School of Public Health, University of Illinois at Chicago; 13 The Dartmouth Institute for Health Policy and Clinical Practice, Norris Cotton Cancer Center, Geisel School of Medicine at Dartmouth, Lebanon, New Hampshire; 14 Division of General Internal Medicine, Massachusetts General Hospital, Harvard Medical School, Boston, Massachusetts; 15 Department of Radiology, Geisel School of Medicine at Dartmouth, Lebanon, New Hampshire; 16 Department of Medicine, University of California, San Francisco; 17 Division of Biostatistics, Department of Public Health Sciences, University of California, Davis, School of Medicine; 18 Kaiser Permanente Washington Health Research Institute, Kaiser Permanente Washington, Seattle

## Abstract

**Importance:**

Digital breast tomosynthesis (DBT) has reduced recall and increased cancer detection compared with digital mammography (DM), depending on women’s age and breast density. Whether DBT screening access and use are equitable across groups of women based on race/ethnicity and socioeconomic characteristics is uncertain.

**Objective:**

To determine women’s access to and use of DBT screening based on race/ethnicity, educational attainment, and income.

**Design, Setting, and Participants:**

This cross-sectional study included 92 geographically diverse imaging facilities across 5 US states, at which a total of 2 313 118 screening examinations were performed among women aged 40 to 89 years from January 1, 2011, to December 31, 2017. Data were analyzed from June 13, 2019, to August 18, 2020.

**Exposures:**

Women’s race/ethnicity, educational level, and community-level household income.

**Main Outcomes and Measures:**

Access to DBT (on-site access) at time of screening by examination year and actual use of DBT vs DM screening by years since facility-level DBT adoption (≤5 years).

**Results:**

Among the 2 313 118 screening examinations included in the analysis, the proportion of women who had DBT access at the time of their screening appointment increased from 11 558 of 354 107 (3.3%) in 2011 to 194 842 of 235 972 (82.6%) in 2017. In 2012, compared with White women, Black (relative risk [RR], 0.05; 95% CI, 0.03-0.11), Asian American (RR, 0.28; 95% CI, 0.11-0.75), and Hispanic (RR, 0.38; 95% CI, 0.18-0.80) women had significantly less DBT access, and women with less than a high school education had lower DBT access compared with college graduates (RR, 0.18; 95% CI, 0.10-0.32). Among women attending facilities with both DM and DBT available at the time of screening, Black women experienced lower DBT use compared with White women attending the same facility (RRs, 0.83 [95% CI, 0.82-0.85] to 0.98 [95% CI, 0.97-0.99]); women with lower educational level experienced lower DBT use (RRs, 0.79 [95% CI, 0.74-0.84] to 0.88 [95% CI, 0.85-0.91] for non–high school graduates and 0.90 [95% CI, 0.89-0.92] to 0.96 [95% CI, 0.93-0.99] for high school graduates vs college graduates); and women within the lowest income quartile experienced lower DBT use vs women in the highest income quartile (RRs, 0.89 [95% CI, 0.87-0.91] to 0.99 [95% CI, 0.98-1.00]) regardless of the number of years after facility-level DBT adoption.

**Conclusions and Relevance:**

In this cross-sectional study, women of minority race/ethnicity and lower socioeconomic status experienced lower DBT access during the early adoption period and persistently lower DBT use when available over time. Future efforts should address racial/ethnic, educational, and financial barriers to DBT screening.

## Key Points

**Question:**

Do access to and use of digital breast tomosynthesis (DBT) screening differ based on women’s sociodemographic characteristics?

**Findings:**

In this cross-sectional study involving 2 313 118 screening examinations performed at 92 Breast Cancer Surveillance Consortium facilities from 2011 to 2017, women of minority race/ethnicity, lower educational attainment, and lower income were less likely to attend a facility offering screening DBT and to obtain DBT rather than digital mammography when both modalities were available at the time of imaging.

**Meaning:**

These findings suggest that women of minority race/ethnicity and lower socioeconomic status experience lower DBT screening access and/or use, especially during the early technology adoption period, suggesting potential widening in persistent breast cancer screening disparities.

## Teaser

This cross-sectional study evaluates women’s access to and use of digital breast tomosynthesis screening based on race/ethnicity, educational attainment, and income.

## Introduction

Routine breast cancer screening with digital breast tomosynthesis (DBT, or 3-dimensional mammography) may improve screening outcomes over traditional digital mammography (DM, or 2-dimensional mammography).^1,2,3^ Multiple prospective trials and observational studies^4,5,6,7,8,9^ demonstrate that DBT can improve the cancer detection rate while decreasing the recall rate from screening compared with DM screening at the population level depending on women’s age, breast density, and screening interval. Since its approval by the US Food and Drug Administration in 2011, DBT has become available in more than two-thirds of all US mammography screening facilities.^10^

Although diffusion of DBT screening in the US has been relatively rapid, it is unknown whether adoption has occurred equally across different populations.^11^ Populations with traditional disparities—Black race, Hispanic ethnicity, lower educational level, or lower income level—have historically experienced greater breast cancer morbidity and mortality than their less disadvantaged counterparts.^12,13,14^ These populations have also historically been the last to benefit from newer medical technologies.^15,16^

To offer DBT screening, imaging facilities must purchase newer mammography units or add additional hardware to make existing units DBT capable.^17^ Thus, not all facilities are able to adopt DBT or immediately offer it to their entire screening population owing to large, up-front equipment costs. Moreover, many insurance companies do not reimburse for DBT screening, forcing women to pay out-of-pocket costs for DBT or opt to obtain only DM screening. This variability in facility-level adoption and individual-level availability may affect access to and use of DBT over DM screening for populations with traditional disparities.

In this analysis, we aimed to determine whether women of minority race/ethnicity and lower socioeconomic status were (1) less likely to have on-site access to DBT screening from 2011 to 2017 and (2) less likely to use DBT over DM screening within facilities offering both modalities at the time of their screening examinations. We hypothesized that these traditionally underserved populations have less access to and persistently lower use of DBT screening. Our study informs breast cancer screening stakeholders, including policy makers and public health advocates, about disparities in access to and use of this newer breast cancer screening imaging modality in community practice.

## Methods

### Study Participants and Setting

Our study cohort included all routine screening mammography examinations performed among women aged 40 to 89 years attending Breast Cancer Surveillance Consortium (BCSC)–affiliated imaging facilities from January 1, 2011, to December 31, 2017. The US Food and Drug Administration approved DBT as a primary screening tool in 2011, at the start of our study period.^17^ The BCSC represents a geographically diverse set of US imaging facilities that collect and pool breast imaging data from a screening population that is representative of the general US population based on women’s age and race/ethnicity.^18^ The BCSC registries contributing data for this analysis included the Carolina Mammography Registry, Metro Chicago Breast Cancer Registry, New Hampshire Mammography Network, San Francisco Mammography Registry, and Vermont Breast Cancer Surveillance System.

All procedures were compliant with the Health Insurance Portability and Accountability Act, and each registry and the BCSC Statistical Coordinating Center received institutional review board approval for a waiver of consent for facility enrollment, data linkage, and analyses. Registries and the BCSC Statistical Coordinating Center also received a federal certificate of confidentiality and other protections for the identities of women, physicians, and facilities. This study followed the Strengthening the Reporting of Observational Studies in Epidemiology (STROBE) reporting guideline.

### Measures

We excluded examinations not meeting our strict definition for routine screening.^19^ All BCSC facilities record clinical indication and modality for each imaging examination, allowing for capture of the on-site availability of screening modalities and their eventual use (DBT or DM). Outcomes included (1) attendance at a facility with on-site DBT availability (hereinafter referred to as DBT access), and (2) use of DBT over DM when both were available at the screening facility (hereinafter referred to as DBT use). Most screening facilities have multiple mammography units, and most facilities adopt DBT in an incremental fashion (replacing 1 mammography unit at a time given the associated large capital expenses). To evaluate actual DBT use when a choice was available, we excluded examinations performed at facilities that offered only DM screening or only DBT screening at the time of imaging.

Our main exposure variables were a woman’s race/ethnicity, educational level, and median household income. For DBT access, we also examined associations with rural residence. At the time of each screening examination, women’s age, residential zip code, self-reported race/ethnicity, and self-reported educational level were recorded (the latter was not collected at 1 registry). We categorized women’s age into decades (40-49, 50-59, 60-69, and ≥70 years). Self-reported racial/ethnic categories included non-Hispanic/Latinx White (hereinafter referred to as White), non-Hispanic/Latinx Black (hereinafter referred to as Black), Asian American, Hispanic/Latinx (hereinafter referred to as Hispanic), and other (Native Hawaiian or Pacific Islander, American Indian or Alaska Native, and mixed race). Educational categories included less than high school, high school diploma (or General Educational Development test passed), some college or technical school, and college degree or higher. We assigned examination-level rural/urban residence status and median household income based on women’s zip code and 2010 Census zip code–level data. Census-based median household income levels were categorized into quartiles for the study population. The most up-to-date US Census rural-urban commuting area data were used to categorize type of residence as urban focused, large rural, small rural, or isolated rural.^20^

### Statistical Analysis

Data were analyzed from June 13, 2019, to August 18, 2020. We report descriptive statistics at the examination level and unadjusted trends of access to and use of DBT at screening over time. To evaluate trends in DBT access over time, we calculated proportions of examinations at facilities after on-site DBT availability by examination year. To evaluate trends in DBT use, we calculated proportions of DBT use by time since facility-level DBT adoption because facilities have increasing DBT availability as older DM mammography units are replaced one by one with DBT-capable mammography units over time.

To evaluate the association between each exposure and DBT screening access, we fit log-binomial regression models adjusting for the correlation within women and facilities using a 3-step generalized estimated equations approach.^21,22^ We included a 2-way interaction term between each exposure and calendar year to evaluate whether any disparities changed over time. For models evaluating actual DBT use when available at the time of screening, we fit log-binomial regression models for race/ethnicity, educational level, and income level separately within each year after facility-level DBT adoption. We included facility as a fixed effect to estimate whether screening examinations from the same facility were more or less likely to be DBT compared with DM based on each exposure. For both outcomes, relative risks (RRs) for each calendar year or year after DBT adoption were estimated, and 95% CIs were computed based on robust covariance matrices. All analyses were performed using SAS, version 9.4 (SAS Institute, Inc). Statistical tests used a 2-sided α = .05; however, we focused on effect sizes and 95% CIs rather than *P* values.

## Results

**Figure 1.**
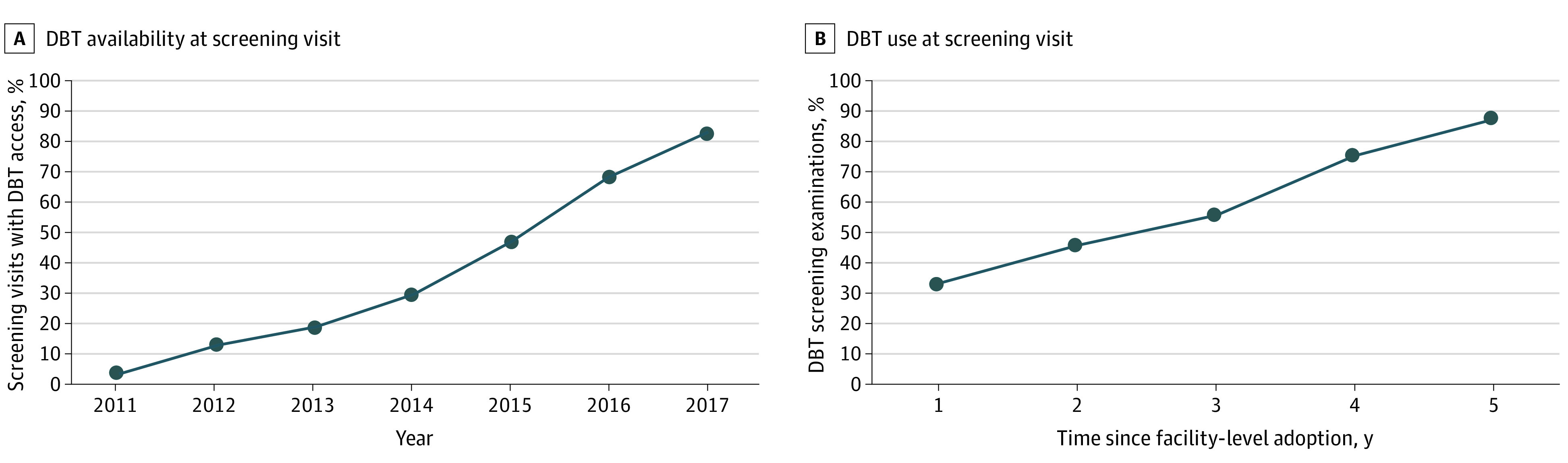
Access to and Use of Digital Breast Tomosynthesis (DBT) at Screening Visit Over Time A, Calculated from 2 313 118 screening examinations included in the analysis. B, Calculated from 631 800 screening examinations in which DBT was obtained over digital mammography at time of imaging by years since facility-level DBT adoption.

A total of 2 313 118 screening examinations from 2011 to 2017 were performed across 92 geographically diverse US screening facilities. Overall, 2 019 352 examinations (87.3%) were from practices in community settings not affiliated with academic institutions. In 2011, 11 558 of 354 107 examinations (3.3%) were performed at facilities with DBT availability compared with 194 842 of 235 972 examinations (82.6%) in 2017 (Figure 1A). During the study period, 41 facilities offered both DBT and DM at the time of screening, performing a total of 631 800 (311 538 DBT and 320 262 DM) screening examinations. Within 1 year of facility-level DBT adoption, 73 091 of 223 535 examinations (32.7%) performed at dual modality facilities were DBT compared with 32 777 of 37 384 examinations (87.7%) within 5 years of facility-level DBT adoption (Figure 1B).

### Screening DBT On-site Access

Overall, 542 181 of 1 459 374 examinations (37.2%) for White women were performed at facilities offering DBT screening compared with 44 371 of 268 773 (16.5%) for Asian American women, 45 917 of 150 258 (30.6%) for Hispanic women, and 104 404 of 303 696 (34.4%) for Black women (Table 1). Examinations for women with a college education were more likely to be performed at facilities offering DBT than for women with less than a high school education (143 137 of 562 003 [25.5%] vs 16 172 of 98 604 [16.4%]). Examinations for women from urban residences were more likely to be performed at facilities offering DBT compared with women from small rural residences (609 356 of 1 789 374 [34.1%] vs 21 126 of 78 744 [26.8%]).

**Table 1.** On-Site Access to and Use of DBT Screening by Sociodemographic Characteristics

Characteristic	No. (%) of participants^a^		
	Total study population (n = 2 313 118)	DBT available at screening visit (n = 778 781)	DBT used if available at screening visit (n = 311 538)
Age, y			
40-49	560 493 (24.2)	181 203 (32.3)	82 403 (55.7)
50-59	726 674 (31.4)	245 462 (33.8)	100 527 (50.4)
60-69	630 099 (27.2)	215 956 (34.3)	82 360 (47.3)
70-89	395 852 (17.1)	136 160 (34.4)	46 248 (41.9)
Race/ethnicity			
White, non-Hispanic	1 459 374 (63.1)	542 181 (37.2)	229 744 (53.1)
Black, non-Hispanic	303 696 (13.1)	104 404 (34.4)	35 923 (37.7)
Hispanic	150 258 (6.5)	45 917 (30.6)	16 743 (44.0)
Asian American	268 773 (11.6)	44 371 (16.5)	13 804 (42.8)
Other	131 017 (5.7)	14 050 (10.7)	5319 (49.8)
Self-reported educational level			
Less than high school diploma	98 604 (8.7)	16 172 (16.4)	5297 (40.8)
High school diploma	220 854 (19.4)	56 802 (25.7)	20 263 (47.7)
Some college	253 955 (22.4)	63 067 (24.8)	22 644 (49.6)
College degree	562 003 (49.5)	143 137 (25.5)	49 722 (50.6)
Geocoded income quartile^b^			
1 (lowest)	517 907 (25.0)	164 540 (31.8)	60 629 (43.9)
2	502 522 (24.2)	211 110 (42.0)	73 246 (50.9)
3	530 648 (25.6)	173 755 (32.7)	74 224 (52.4)
4 (highest)	521 610 (25.2)	150 150 (28.8)	72 949 (51.4)
Geocoded residence			
Urban focused	1 789 374 (85.4)	609 356 (34.1)	255 918 (49.6)
Large rural	131 761 (6.3)	42 592 (32.3)	13 235 (46.5)
Small rural	78 744 (3.8)	21 126 (26.8)	6181 (64.4)
Isolated rural	95 157 (4.5)	32 398 (34.0)	7804 (50.0)

Abbreviation: DBT, digital breast tomosynthesis.

^a^ Numbers do not sum to some total sample sizes owing to missingness. Examinations with DBT available are calculated as percentage of row total. Examinations with DBT used are calculated as percentage of those with DBT available.

^b^ Calculated as income quartiles observed in the Breast Cancer Surveillance Consortium study population. For DBT access, quartile 1 indicates less than $60 781; quartile 2, $60 781 to $77 663; quartile 3, $77 664 to $99 953; and quartile 4, greater than $99 953. For DBT use, quartile 1 indicates less than $61 155; quartile 2, $61 155 to $74 904; quartile 3, $74 905 to $93 952; and quartile 4, greater than $93 952.

**Figure 2.**
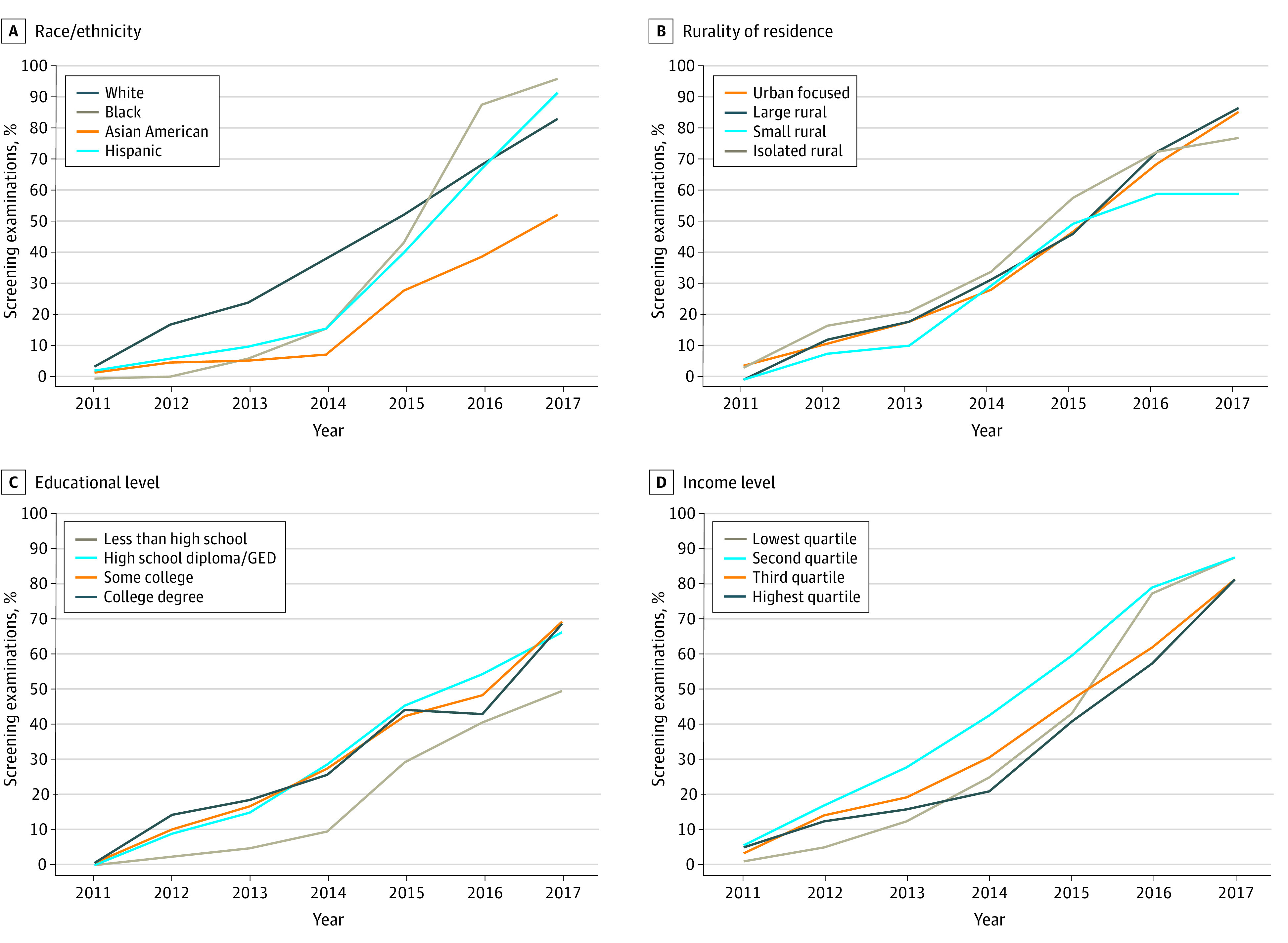
Digital Breast Tomosynthesis (DBT) Access at Screening Visit by Sociodemographic Characteristics Over Time Unadjusted proportions of screening examinations in 2011 to 2017 for which DBT was available at the facility level at time of imaging are calculated based on year and race/ethnicity (A), rurality of residence (B), educational level (C), and cohort quartile of zip code–based median household income (D). GED indicates General Educational Development.

Unadjusted trends in DBT access over time based on race/ethnicity, educational level, income, and rural residence are depicted in Figure 2. Asian American, Black, and Hispanic women experienced lower DBT access in earlier years compared with White women (ie, 5.5% of Asian American, 6.6% of Black, and 10.0% of Hispanic vs 24.0% of White women in 2013), but this gap closed by 2017 except for Asian American women (52.0% of Asian American vs 82.8% of White women in 2017). Similarly, women with a lower educational level, women living in zip codes with lower median household income, and women with rural residence experienced lower DBT access in early study years, with convergence by the end of the study period except for women with less than a high school education (49.5% compared with 69.0% of college graduates in 2017) and rural residence (59.1% for small rural and 77.1% for isolated rural residences compared with 85.4% of urban residence in 2017).

In multivariable analyses, on-site DBT access at time of imaging differed based on women’s race/ethnicity and educational level in early study years (Table 2). In 2012, for instance, Black (RR, 0.05; 95% CI, 0.03-0.11), Asian American (RR, 0.28; 95% CI, 0.11-0.75), and Hispanic (RR, 0.38; 95% CI, 0.18-0.80) women had significantly lower on-site access compared with White women; women with less than a high school education (RR, 0.18; 95% CI, 0.10-0.32), a high school diploma (RR, 0.62; 95% CI, 0.42-0.91), and some college (RR, 0.71; 95% CI, 0.57-0.89) had lower DBT access compared with college graduates. However, by 2017, DBT access did not significantly differ based on women’s race/ethnicity or educational level.

**Table 2.** Unadjusted Relative Risks of Digital Breast Tomosynthesis Screening On-site Access by Sociodemographic Characteristics

Characteristic by examination year	On-site DBT access, RR (95% CI)		
**Race/ethnicity**^**a**^	**Black**	**Hispanic**	**Asian American**
2011	0.06 (0.04-0.11)	0.64 (0.42-0.97)	0.47 (0.25-0.92)
2012	0.05 (0.03-0.11)	0.38 (0.18-0.80)	0.28 (0.11-0.75)
2013	0.27 (0.09-0.82)	0.42 (0.22-0.81)	0.23 (0.09-0.57)
2014	0.42 (0.18-1.00)	0.42 (0.22-0.81)	0.21 (0.09-0.47)
2015	0.84 (0.57-1.23)	0.77 (0.57-1.04)	0.54 (0.27-1.07)
2016	1.28 (1.05-1.56)	0.98 (0.77-1.25)	0.57 (0.30-1.05)
2017	1.16 (1.02-1.32)	1.10 (0.98-1.24)	0.63 (0.34-1.17)
**Educational level**^**b**^	**Less than high school**	**High school diploma**	**Some college**
2011	0.24 (0.14-0.40)	0.22 (0.15-0.31)	0.76 (0.62-0.93)
2012	0.18 (0.10-0.32)	0.62 (0.42-0.91)	0.71 (0.57-0.89)
2013	0.26 (0.13-0.53)	0.82 (0.50-1.36)	0.90 (0.65-1.23)
2014	0.38 (0.19-0.74)	1.10 (0.69-1.75)	1.06 (0.78-1.42)
2015	0.66 (0.38-1.14)	1.02 (0.71-1.48)	0.95 (0.78-1.17)
2016	0.94 (0.44-1.99)	1.26 (0.94-1.70)	1.12 (0.96-1.32)
2017	0.72 (0.33-1.54)	0.96 (0.76-1.21)	1.00 (0.89-1.13)
**Geocoded income**^**c**^	**BCSC quartile 1**	**BCSC quartile 2**	**BCSC quartile 3**
2011	0.08 (0.04-0.14)	1.10 (0.64-1.88)	0.63 (0.46-0.86)
2012	0.40 (0.11-1.51)	1.40 (0.71-2.76)	1.16 (0.48-2.80)
2013	0.79 (0.25-2.54)	1.76 (0.88-3.52)	1.21 (0.61-2.39)
2014	1.19 (0.48-2.97)	2.05 (1.06-3.96)	1.46 (0.83-2.56)
2015	1.06 (0.61-1.82)	1.46 (0.93-2.30)	1.16 (0.84-1.59)
2016	1.35 (0.95-1.91)	1.38 (1.01-1.89)	1.08 (0.85-1.39)
2017	1.08 (0.91-1.28)	1.08 (0.92-1.26)	1.00 (0.89-1.13)
**Geocoded residence**^**d**^	**Large rural**	**Small rural**	**Isolated rural**
2012	1.07 (0.15-7.64)	0.70 (0.17-2.87)	1.49 (0.30-7.47)
2013	1.00 (0.25-4.07)	0.56 (0.18-1.77)	1.16 (0.33-4.07)
2014	1.09 (0.45-2.63)	1.04 (0.45-2.42)	1.20 (0.53-2.73)
2015	0.99 (0.48-2.02)	1.05 (0.53-2.11)	1.24 (0.74-2.08)
2016	1.06 (0.73-1.52)	0.86 (0.45-1.67)	1.06 (0.69-1.63)
2017	1.02 (0.80-1.29)	0.69 (0.36-1.34)	0.90 (0.63-1.31)

Abbreviations: BCSC, Breast Cancer Surveillance Consortium; DBT, digital breast tomosynthesis; RR, relative risk.

^a^ Reference group consists of White women.

^b^ Reference group consists of college degree.

^c^ Reference group consists of BCSC quartile 4. Calculated as income quartiles observed in BCSC study population. For DBT access, quartile 1 indicates less than $60 781; quartile 2, $60 781 to $77 663; quartile 3, $77 664 to $99 953; and quartile 4, greater than $99 953.

^d^ Reference group consists of urban residence. Geocoded residence is not portrayed for 2011 because all DBT examinations that year were from a single regional registry.

### Screening DBT Use

Overall, Black (35 923 of 95 411 [37.7%]), Hispanic (16 743 of 38 067 [44.0%]), and Asian American (13 804 of 32 222 [42.8%]) women were less likely to obtain DBT vs DM screening when both were available at the time of imaging compared with White women (229 744 of 432 549 [53.1%]) (Table 1). Women with lower educational levels (5297 of 12 968 [40.8%] with less than a high school education vs 49 722 of 98 170 [50.6%] with a college degree) and living in zip codes with lower median household income (60 629 of 137 969 [43.9%] for the lowest income quartile vs 72 949 of 141 807 [51.4%] for the highest income quartile) were also less likely to obtain DBT when both DBT and DM were available.

Unadjusted rates of DBT vs DM use over time based on race/ethnicity, educational level, and income are depicted in the eFigure in the Supplement. Black women had persistently lower rates of DBT use up to 4 years after facility-level DBT adoption compared with White women (27.9% vs 35.6% within 1 year, 40.6% vs 48.0% within 2 years, 41.9% vs 59.4% within 3 years, and 58.0% vs 77.2% within 4 years after DBT adoption). Women with less than a high school education persistently had lower DBT use compared with college graduates 5 years after DBT adoption (66.9% of those with less than a high school education vs 87.3% of college graduates). Women living in zip codes with lower median household income had lower DBT use up to 4 years after facility-level DBT adoption (62.6% for the lowest vs 81.7% for the highest income quartiles 4 years after DBT adoption).

**Figure 3.**
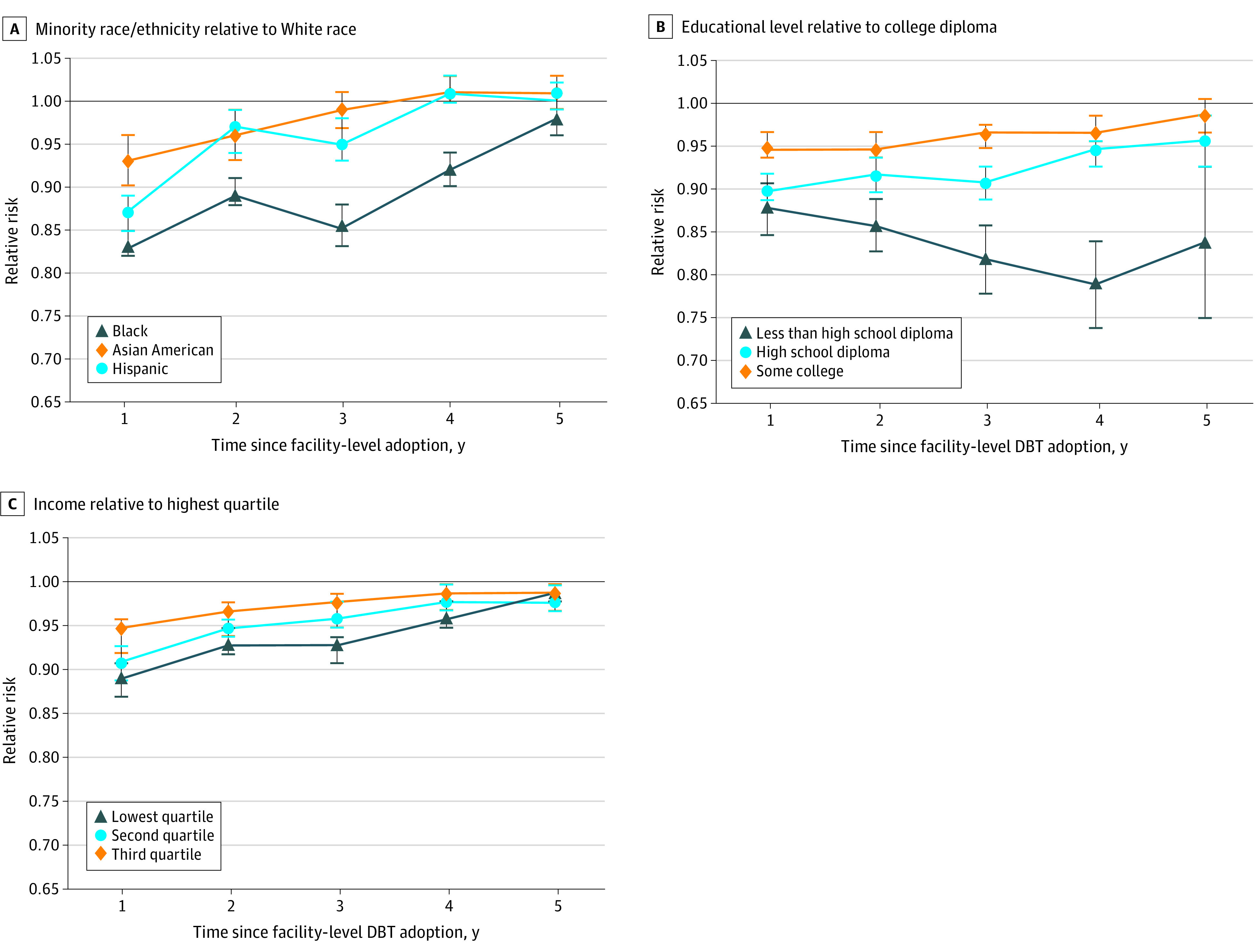
Digital Breast Tomosynthesis (DBT) Use If Available During the Screening Visit by Race/Ethnicity, Educational Level, and Income Level Over Time Data are calculated as relative risk of obtaining DBT over digital mammography when both modalities were available at time of screening by race/ethnicity (A), educational level (B), and income quartile (C) and by year since facility-level DBT adoption. Error bars indicate 95% CIs.

In multivariable analyses, the use of DBT compared with DM screening differed within the same facilities based on women’s race/ethnicity, educational level, and income for multiple years after facility-level DBT adoption (Figure 3). Within 1 year of DBT adoption, Black women had an RR of 0.83 (95% CI, 0.82-0.85), and Hispanic women had an RR of 0.87 (95% CI, 0.85-0.89) for DBT screening compared with White women within the same facility. Four years after DBT adoption, Black women continued to have a lower RR of 0.92 (95% CI, 0.90-0.94) for DBT screening use compared with White women attending the same facility. Women with lower educational levels were less likely to use DBT over DM regardless of the number of years after facility-level DBT adoption (RRs, 0.79 [95% CI, 0.74-0.84] to 0.88 [95% CI, 0.85-0.91] for non–high school graduates and 0.90 [95% CI, 0.89-0.92] to 0.96 [95% CI, 0.93-0.99] for high school graduates vs college graduates). Similarly, women within the lowest income quartile were less likely to use DBT over DM regardless of the number of years after facility-level DBT adoption (RRs, 0.89 [95% CI, 0.87-0.91] to 0.99 [95% CI, 0.98-1.00]).

## Discussion

Our large cross-sectional study provides evidence of disparities in the diffusion of DBT screening technology in US practices. From 2011 to 2017, the proportion of screening examinations performed at BCSC facilities offering DBT screening increased rapidly from 3.3% to 82.6%; however, technology adoption was unequal based on race/ethnicity and socioeconomic characteristics of the patients. Women of minority race/ethnicity and with lower educational attainment, lower household income, and rural residence were less likely to have access to DBT screening for years after the 2011 US Food and Drug Administration approval of DBT and were less likely to undergo DBT screening even when it became widely available.

On-site access to DBT improved for most sociodemographic groups over time as more facilities incrementally replaced DM with DBT screening, especially with regard to neighborhood income levels. However, Asian American women, women with less than a high school education, and those living in small rural settings had persistently lower DBT access at the end of our study period. The disparities based on educational level and rural residence are in line with prior BCSC analyses that demonstrated that other advanced imaging technologies, such as breast magnetic resonance imaging, are less accessible to US women with rural residence and lower educational levels.^23,24^

Even as on-site access to DBT improved over time, our analysis demonstrates disparities in DBT use based on socioeconomic characteristics. Women living in zip codes with lower income quartiles and women with less than a high school education had lower DBT compared with DM use throughout the study period. In previous analyses,^25,26^ we found that the ability to pay out-of-pocket costs associated with newer screening technologies and knowledge of benefits vs harms of new screening technologies influenced their use. Medicare did not reimburse for DBT until 2015, and third-party payer coverage for DBT remains payer dependent, which suggests that insurance coverage and copayments may be an important factor for trends in DBT use observed in our data. Our findings regarding socioeconomic status are further supported by a claims analysis of an insured population in 2015 to 2017 that demonstrated more DBT use in geographic areas with higher income and educational attainment.^27^ The identification of a strong association of lower educational attainment and income with consistently lower DBT use provides a basis for novel interventions, including addressing financial barriers, such as copayments or out-of-pocket costs associated with DBT screening at the facility level.^28^

With regard to racial/ethnic disparities in DBT use, Black women were consistently less likely to obtain DBT over DM when both modalities were available. The gap in DBT use between Black vs White women persisted regardless of the number of years after facility-level DBT adoption. This finding is important, especially given the higher risk of advanced-stage breast cancer at the time of diagnosis among Black women relative to White women.^12,15^ Persistent differences in DBT use between Black and White women may suggest a widening of cancer screening disparities, because DBT is associated with improved outcomes in terms of both improved cancer detection and lower false-positive rates for some subgroups of women.^1,8,9,11,29,30^

### Strengths and Limitations

Strengths of this study include its large sample size of 2 313 118 screening examinations and geographic diversity in the 92 imaging facilities in 5 different states. Our results support the general hypothesis that women of minority race/ethnicity and lower socioeconomic status tend to be the last to adopt and benefit from newer medical technologies, including novel interventional cardiology, spine, and colorectal cancer screening procedures.^15,16,31^ However, the study has limitations. First, the BCSC captures data only from women who obtain breast imaging, and thus we cannot examine disparities in DBT vs DM use among women who do not undergo routine screening mammography. Second, it was not possible for us to examine facility-level workflow processes that may have influenced selection of women for DBT vs DM screening at the time of their imaging (eg, women with dense breasts may have been preferentially scheduled for DBT screening at some facilities, whereas other facilities may schedule women for the first available mammography machine).^32^ Third, we did not account for individual breast cancer risk factors, such as breast density or family history, because a prior analysis^9^ demonstrated that use of DBT vs DM did not widely differ based on these risk factors. Finally, the BCSC does not have complete ascertainment of insurance status, preventing us from examining this as an exposure. The DBT screening examination, although covered by Medicare as of 2015, is likely associated with additional out-of-pocket costs for some patients.^28,33^ To address this limitation, we used US Census–based geocoded income quartiles as a surrogate for potential financial barriers.

## Conclusions

In summary, this cross-sectional study found that women of minority race/ethnicity and lower socioeconomic status have lower DBT screening access and use in the current mixed-modality environment for breast cancer screening, especially in the years immediately after a facility’s DBT adoption. Black women, women with lower educational attainment, and women with lower incomes continue to experience disparities in obtaining DBT compared with DM at mixed-modality screening facilities, even years after facility-level DBT adoption. Although most mammography facilities in the US now offer DBT screening on at least 1 of their mammography units, only about 40% of all certified mammography units in the US were DBT capable as of August 2020.^10^ Thus, radiology practices and policy makers should be cognizant of these screening access and use differences, and future efforts should address racial/ethnic, educational, financial, and geographic barriers to obtaining DBT screening at the facility level.

## Notes

Supplement. **eFigure.** DBT Use at Dual Modality Facilities by Sociodemographic Characteristics Over Time
