# Supplementary material for: Comparative Access to and Use of Digital Breast Tomosynthesis Screening by Women’s Race/Ethnicity and Socioeconomic Status
Source: JAMA Netw Open. 2021 Feb 19;4(2):e2037546. doi: 10.1001/jamanetworkopen.2020.37546 (PMC7896194; doi:10.1001/jamanetworkopen.2020.37546)
Supplement: Supplement. — eFigure. DBT Use at Dual Modality Facilities by Sociodemographic Characteristics Over Time [file jamanetwopen-e2037546-s001.pdf]

# Supplementary Online Content

Lee CI, Zhu W, Onega T, et al. Comparative access to and use of digital breast tomosynthesis screening by women’s race/ethnicity and socioeconomic status. *JAMA Netw Open*. 2021;4(2):e2037546. doi:10.1001/jamanetworkopen.2020.37546

**eFigure.** DBT Use at Dual Modality Facilities by Sociodemographic Characteristics Over Time

This supplementary material has been provided by the authors to give readers additional information about their work.

©2021 Lee CI et al. *JAMA Network Open*.

**eFigure. DBT Use at Dual Modality Facilities by Sociodemographic Characteristics Over Time**

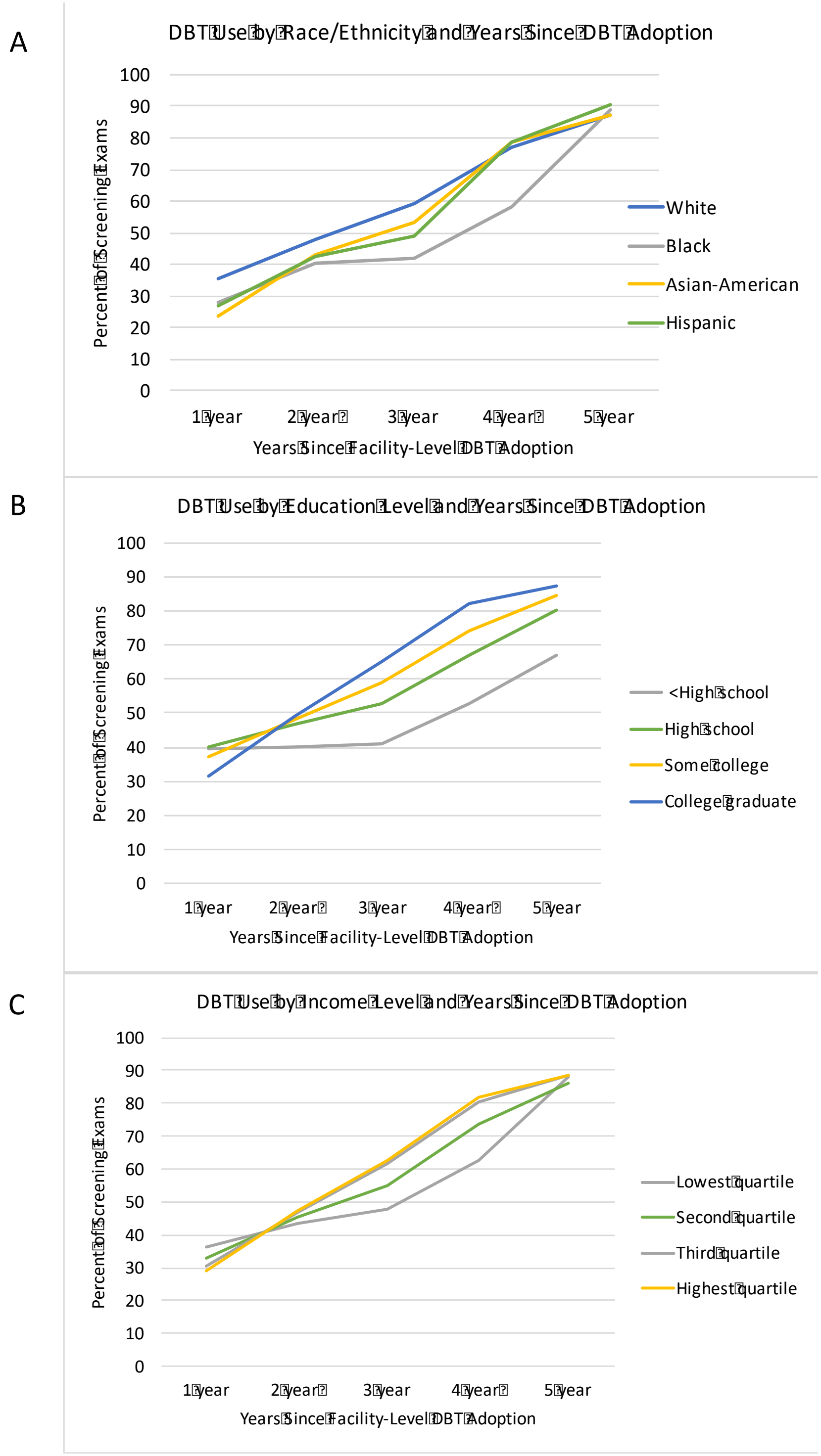


**eFigure 1.** Unadjusted proportions of screening exams in 2011-2017 that were DBT at facilities offering both DM and DBT screening at time of imaging based on race/ethnicity (A), education level (B), and quartile of zip code-based median household income (C).

©2021 Lee CI et al. *JAMA Network Open*.
